# Hip Fractures Sustained Through Inpatient Falls Are Associated With Lower Rates of Fascia Iliaca Block Administration and Higher Opiate Requirements: A Single-Centre Experience

**DOI:** 10.7759/cureus.103242

**Published:** 2026-02-08

**Authors:** Hannah Jeal, Matt Towner, Joshua Moreau, Sally Rankin, Jonathan French, Michael Kelly

**Affiliations:** 1 Department of Trauma and Orthopaedics, Southmead Hospital, Bristol, GBR; 2 Department of Trauma and Orthopaedics, John Radcliffe Hospital, Oxford, GBR

**Keywords:** fascia iliaca compartment block, inpatient falls, neck of femur fractures, peri-operative analgesia, perioperative pain management

## Abstract

Background

Hip fractures, also known as neck of femur (NOF) fractures, are a major cause of morbidity in older adults. Effective pain control is essential to minimise opioid-related complications such as delirium. Fascia iliaca blocks (FIBs) provide regional anaesthesia that can reduce opioid requirements. While emergency department (ED) pathways routinely include FIBs for NOF patients, it is unclear whether those who sustain NOFs during inpatient admissions receive comparable care.

Methods

A retrospective case-control study was conducted at a single NHS major trauma centre between January 2019 and December 2020. Patients aged over 60 with radiologically confirmed NOFs sustained during an inpatient stay were compared with age-, sex-, American Society of Anesthesiologists (ASA) grade-, premorbid mobility-, and Abbreviated Mental Test Score (AMTS)-matched controls admitted via the ED, irrespective of FIB status. Primary outcomes were administration of a FIB and 24-hour opioid use (oral morphine equivalent dose). Secondary outcomes were postoperative delirium, postoperative length of stay, and all-cause mortality at 30 days and one year.

Results

Of 4026 inpatient falls, 36 resulted in NOFs (3% of all NOFs during the study period (36/1182)). Only 17% (6/36) of inpatients received a FIB compared with 86% (988/1146) of ED-admitted patients (P<0.001). Patients who received a FIB had significantly lower 24-hour opioid requirements (mean 17.3 mg vs. 35.4 mg oral morphine equivalent doses (OMED); P=0.037). Postoperative delirium occurred in 20% of non-FIB patients versus 11.9% in the FIB group (P=0.347). Mean postoperative length of stay was longer for inpatients than for matched ED controls (41.5 vs. 15.2 days; P=0.01). Mortality at 30 days and one year did not differ significantly between groups.

Conclusions

Inpatients sustaining NOFs were markedly less likely to receive a FIB than ED-admitted patients and had higher opioid requirements and longer hospital stays. These findings highlight a disparity in pain management between inpatient and ED pathways and support the development of hospital-wide protocols to ensure timely regional anaesthesia for all NOF patients.

## Introduction

Fractured neck of femur (NOF) constitutes a significant health burden in ageing populations. The incidence of NOFs rises exponentially with age due to increasing frailty, osteoporosis, and falls risk [1]. Hip fractures represent a significant burden to the UK National Health Service (NHS), with over 75,000 cases annually and an estimated cost of £2 billion per year [2].

These injuries are acutely painful, often necessitating high-dose opioid analgesia [3,4]. However, older adults are particularly vulnerable to opioid-induced complications, including delirium - a condition associated with poor functional outcomes, prolonged hospitalisation, and increased mortality [5-8]. Notably, effective pain control is a modifiable risk factor in delirium prevention; improved analgesia can reduce the risk of delirium by as much as ninefold [9].

Fascia iliaca blocks (FIBs) offer a regional anaesthetic technique targeting the femoral, lateral femoral cutaneous and obturator nerves via local anaesthetic administration beneath the fascia iliaca [10]. FIBs reduce pain scores and opioid use [4,11]. FIBs have been shown to decrease the incidence of delirium in NOF patients without underlying cognitive impairment [12]. They have also been associated with decreased pain on movement, reduced time to mobilisation, decreased incidence of pneumonia, and lower overall analgesia costs [13]. FIBs are recommended by the National Institute for Health and Care Excellence as part of routine hip fracture management, as well as by the Royal College of Emergency Medicine [14,15]. 

Inpatient falls, leading to NOFs, comprise approximately 3% of all hip fractures [2,16]. Whilst patients admitted after neck of femur fracture in the community routinely receive FIBs, the same cannot be guaranteed for patients sustaining NOFs in-hospital. These patients bypass the ED and may thus experience deviation from protocolised pathways, including omission of FIBs, as FIB administration within the ED is carried out primarily by ED resident doctors. This disparity could expose inpatients to a higher opioid burden and the complications associated with this. 2023 data suggest that up to 52% of patients sustaining in-hospital NOFs develop delirium - more than three times the incidence reported in ED-managed cohorts [8,17]. 

This study assessed the rate of FIB administration in patients sustaining a NOF by falling as an inpatient at an NHS major trauma centre, and whether this was associated with increased opioid use, delirium, and mortality. We hypothesised that patients with NOFs sustained during inpatient falls would less frequently be given a FIB for analgesia, resulting in poorer outcomes.

## Materials and methods

Study design and data sources

This retrospective case-control study was conducted at Southmead Hospital, North Bristol NHS Trust, using data from January 2019 to December 2020. Data sources included the hospital inpatient falls database, the National Hip Fracture Database [16], patient medical records and drug charts accessed via the Electronic Data Management System (EDMS), as well as radiological data accessed via Insight PACS (Insignia Medical Systems). Quality and Safety Improvement Team approved the study (no. QI9930).

Selection criteria

Participants were included in the case group if they were aged over 60 years and had a radiologically confirmed NOF sustained during an inpatient admission. Eligible cases were limited to incidents occurring between January 2019 and December 2020.

The control group comprised patients aged over 60 years who sustained an out-of-hospital NOF and were admitted via ED. Controls were selected using nearest neighbour matching based on age, sex, American Society of Anesthesiologists (ASA) grade [18], premorbid mobility status [19], and Abbreviated Mental Test Score (AMTS) [20].

Exclusion criteria for both groups included patients under the age of 60 and patients who did not have age, sex, ASA, premorbid mobility status or AMTS recorded. 

Outcomes

The primary outcomes of interest in this study were the administration of a FIB and total opioid consumption within the first 24 hours following injury, standardised to oral morphine equivalent doses (OMED) [21]. The secondary outcomes were the incidence of postoperative delirium, defined as a 4AT 4 ‘A’s Test) score of ≥4 within 24 hours following operation; postoperative length of hospital stay (LOS); and all-cause mortality at 30 days and one year.

Delirium was identified using a combination of clinical documentation and validated screening tools recorded in the electronic medical record. To account for potential lead-time bias among the inpatient cohort, LOS was calculated from the date of surgery to the date of discharge, rather than from the date of injury. Mortality data were obtained through institutional records and verified using hospital administrative and national databases to ensure completeness and accuracy.

Statistical analysis 

Continuous variables were expressed as means and standard deviations (SDs); categorical variables were expressed as frequencies and percentages. Between-group comparisons used independent-samples t-tests for continuous data and chi-square tests for categorical data. Matching was done using nearest-neighbour matching on age, sex, ASA grade, prior mobility status and AMTS. Matching was performed without replacement, and no calliper or threshold was applied. Kaplan-Meier survival analysis with log-rank testing was performed for mortality comparison. Statistical significance was defined as P < 0.05.

## Results

Of 4026 total inpatient falls over the study period, 36 resulted in radiologically confirmed NOFs. These accounted for 3% of all NOFs at the centre (36/1182). Among ED-admitted NOF patients, 86% received a FIB (988/1146), compared to only 17% (6/36) of inpatients (P<0.001). All patients with a NOF occurring during the study period were included for FIB status analysis; however, only those occurring as an inpatient, and their matched ED controls, were analysed for outcome data. Table 1 shows the demographics for the inpatient hip fracture patients and their ED-matched controls, demonstrating differences between groups (Table 1).

**Table 1:** Comparison of demographics and FIB status between inpatient and outpatient fall cohorts Table comparing demographics and FIB status of the inpatient fall group and their matched ED-admitted controls. *Chi-square test for categorical data and t-test for continuous data; ^†^Mean (standard deviation) AMTS: Abbreviated Mental Test Score; ASA: American Society of Anesthesiologists grade; ED: Emergency Department; FIB: fascia iliaca block

	Inpatient fall	Matched ED controls	Test Value (P Value*)
	(n=36)	(n=36)	
Sex			
Female	13 (36.1%)	16 (44.4%)	0.471
Male	23 (63.9%)	20 (55.6%)	
Age (Years)^†^	83.11 (6.00)	85.67 (7.55)	0.116
ASA			
III	22 (61.1%)	22 (61.1%)	1.000
IV	14 (38.9%)	14 (38.9%)	
Preoperative AMTS^†^	5.97 (3.88)	5.89 (4.26)	0.939
Mobility			
Freely mobile without aids	10 (27.8%)	8 (22.2%)	0.685
Mobile outdoors with one aid	3 (8.3%)	5 (13.9%)	
Mobile outdoors with two aids or frame	4 (11.1%)	2 (5.6%)	
Mobile indoors only	19 (52.8%)	21 (58.3%)	
Fascia-iliaca block performed			
No	30 (83.3%)	0 (0.0%)	<0.001
Yes	6 (16.7%)	36 (100.0%)	

Patients who received an FIB had a significantly lower 24-hour post-injury opioid requirement (mean 17.3 mg OMED) compared to those who did not (35.4 mg OMED; P=0.037). Compared to FIB patients, postoperative delirium occurrence was almost double that for non-FIB patients (6/30 (20%) versus 5/42 (11.9%)), though this difference was not statistically significant (P=0.347) (Table 2). 

**Table 2:** Patient outcomes according FIB status Table demonstrating patient demographics and outcomes according to FIB status with test value documented. *24-hour opiate requirement, calculated by converting all forms into morphine equivalent in milligrams; **Chi-square test for categorical data and t-test for continuous data; ^†^Mean (standard deviation) LOS: length of hospital stay; FIB: fascia iliaca block

Patient demographics/outcomes	Fascia iliaca block performed		
	No	Yes	Test value (P value)**
	(N=30)	(N=42)	
Sex			
Female	10 (33.3%)	19 (45.2%)	0.310
Male	20 (66.7%)	23 (54.8%)	
Age (years)^†^	82.97 (6.39)	85.40 (7.13)	0.140
Opiate requirement*^†^ (24h; mg)	35.40 (51.38)	17.34 (16.67)	0.040
Postoperative delirium present (number of patients (%))	6 (20.0%)	5 (11.9%)	0.350
Postoperative LOS (days)^†^	36.44 (41.97)	22.72 (42.20)	0.200
30-day mortality (number of patients (%))	7 (23.3%)	11 (26.2%)	0.780
1-year mortality (number of patients (%))	15 (50.0%)	19 (45.2%)	0.690

**Figure 1:**
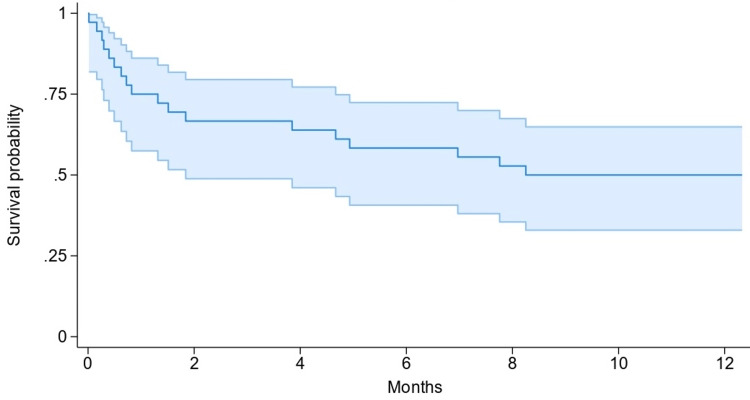
Kaplan–Meier survival curve for mortality in the inpatient NOF cohort Kaplan–Meier survival curve showing pooled survival probability for the inpatient NOF cohort over the 12 months following injury, with 95% confidence intervals shaded. NOF: neck of femur

**Figure 2:**
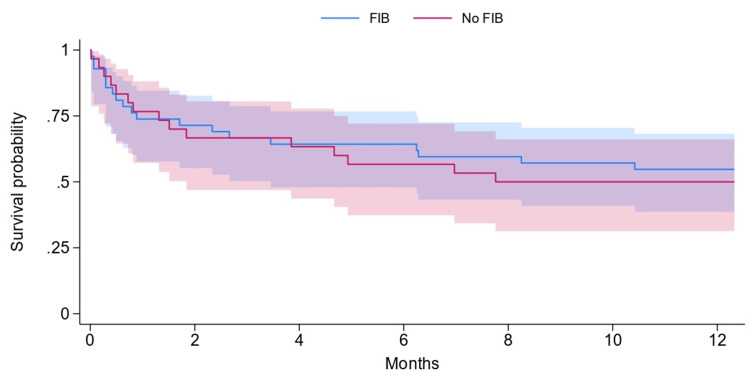
Kaplan–Meier survival curves for mortality according to fascia iliaca block (FIB) status Kaplan–Meier survival analysis with log-rank testing was performed to compare mortality between patients who received an FIB (blue line) and those who did not (red line).  Survival probability up to 12 months is shown for both groups, with 95% confidence intervals shaded.

The average length of stay following surgery was significantly longer for inpatients (41.5 days, SD 56.4) than for matched outpatient falls (15.2 days, SD 10.2; P=0.01) (Table 1). No significant differences were observed in 30-day (26.2% vs. 23.3%) or one-year mortality (45.2% vs. 50%) between FIB and non-FIB groups (P=0.783 and P=0.69, respectively) (Tables 2, 3; Figures 1, 2). The average age of those receiving a FIB was 85.4 years, compared to 82.97 years in those not receiving a FIB. 

**Table 3:** Comparison of the number of patients at risk in Kaplan-Meier survival analysis between cohorts Comparison of the number of patients at risk of death at each time point between the inpatient NOF group and patients analysed based on FIB status. NOF: neck of femur; FIB: fascia iliaca block

Time point (months)	Number at risk in inpatient NOF cohort	Number at risk in patients receiving FIB	Number at risk in patients not receiving FIB
0	36	42	30
2	24	30	20
4	23	27	19
6	21	27	17
8	19	25	15
10	19	24	15
12	18	23	15

## Discussion

Implications

The findings highlight a disparity in NOF pain management between inpatient and ED admissions, suggesting a need to extend established FIB protocols hospital-wide. This could involve ensuring resident doctors on the orthopaedic registrar and senior house officer rota are proficient in administering FIBs when assessing these patients, as well as developing Trust-wide pathways for inpatient NOFs and empowering nursing teams to monitor FIB efficacy and complications. 

The observed reduction in opioid requirements in the FIB cohort aligns with prior literature [22,23], underscoring the role of regional anaesthesia in multimodal pain strategies. Although not statistically significant in our study, the lower incidence of delirium in the FIB group (11.9% vs 20%) parallels prior literature suggesting that strategies reducing opioid exposure and pain may mitigate delirium risk [9,12]. Noteably, in a large ICU cohort, each incremental 20 mg of daily OMED was independently associated with an approximately 2.4% increase in the odds of delirium on the next day [24].

The significantly longer hospital stay among the inpatient-acquired NOF group may reflect the baseline frailty and functional deficits of hospitalised individuals, consistent with other studies showing poorer rehabilitation potential and outcomes in this subgroup [25].

Our institution follows the modern trend of having a high number of single-occupancy rooms compared to bays with multiple beds, with each 32-bed unit consisting of 24 single-occupancy rooms and two four-bed bays [26]. This means that direct supervision of patients who are at risk of falls may be more challenging compared to traditional bay organisation of beds, where a member of staff in the bed areas may have greater potential to opportunistically step in and prevent or decrease the harm associated with a fall. The reduced intensity of observation for the increasing numbers of patients in single rooms makes the importance of robust perioperative pathways for NOF fractures sustained as an inpatient more pressing in order to reduce the risk of further injury associated with opioid-induced delirium [27].

Strengths and limitations

This study has several notable strengths. It utilises real-world, single-centre data with full integration of electronic medical records, allowing for detailed and accurate retrieval of clinical, pharmacological, and outcome data. The control group was well-matched to the inpatient cohort based on key confounders, enhancing the validity of between-group comparisons. Importantly, the study incorporates both process measures, such as the administration of FIBs, and clinically relevant outcomes, including opioid usage, delirium incidence, and mortality. This comprehensive approach facilitates a more nuanced understanding of the relationship between analgesic practice and patient outcomes in NOFs.

However, several limitations must be acknowledged. The sample size was relatively small, particularly within the inpatient fracture cohort, which may limit statistical power and the ability to detect subtle differences between groups. Our findings suggested a trend toward higher opioid requirements in the non-FIB group alongside increased delirium rates; however, this association did not reach statistical significance, likely reflecting limited cohort size and insufficient statistical power. This trend reflects wider literature linking opioid exposure with delirium risk, and suggests that larger studies may be required to fully elucidate the relationship.

Limitations of the retrospective study design include risk of bias, such as unmeasured confounding variables, including intercurrent injury or infection, which may be higher in the inpateint cohort and could independently increase the risk of delirium. Potential inaccuracies in clinical documentation may also reduce the internal validity of the study, with rates of delirium potentially being under-documented. Delirium assessment relied primarily on clinical notes and screening scores, which may underestimate the true incidence due to limited sensitivity compared to formal diagnostic criteria. Finally, as the study was conducted at a single centre, institutional differences in staffing models, analgesic pathways, and procedural availability between inpatient and ED settings may affect the generalisability of these findings to other healthcare settings. Further similar multi-centre studies will be necessary to determine whether the present findings are an isolated phenomenon or a more widespread issue across the NHS and other healthcare systems.

Future work 

To further develop the evidence base surrounding FIB use in NOFs sustained through inpatient falls, several areas of future research are recommended. Large-scale multicentre prospective cohort studies would elucidate experience across diverse hospital settings and NHS trusts. More robust and consistent identification of delirium using standardised validated measures would underpin a clearer understanding of its relationship to analgesic practices. Qualitative research should explore clinician-level barriers to FIB provision in inpatient settings, clarifying practical, educational, or systemic obstacles to the wider adoption of regional anaesthesia outside of ED. Implementation science studies should evaluate the effectiveness and safety of ward-based FIB education and delivery, including the feasibility of expanding training to orthopaedic registrars and senior house officers.

Health economic modelling is needed to assess the cost-effectiveness of implementing universal FIB protocols, particularly in relation to reduced opioid use, delirium incidence, and potential downstream savings from shorter lengths of stay and improved rehabilitation trajectories. These analyses would support resource allocation and policy development at the trust and national levels. 

Mortality outcomes did not differ significantly between groups, but further research with larger sample sizes may be required to detect smaller effect sizes.

## Conclusions

This study provides evidence that inpatients who sustain NOFs are substantially less likely to receive a FIB, despite experiencing equivalent injury severity to their ED-admitted counterparts. The absence of FIB administration in this important and vulnerable subgroup of patients may contribute to elevated opioid use and its associated risks. The findings of this study are in keeping with the wider evidence base and further highlight the need for standardised analgesia protocols for inpatient falls.
